# Supplementary material for: The QseB response regulator imparts tolerance to positively charged antibiotics by controlling metabolism and minor changes to LPS
Source: mSphere. 2023 Sep 7;8(5):e00059-23. doi: 10.1128/msphere.00059-23 (PMC10597456; doi:10.1128/msphere.00059-23)
Supplement: Supplemental Results — Expanded description of RNASeq, supplemental figures, and Table S1. [file msphere.00059-23-s0001.pdf]

Supplementary Information

Supplementary Results:

Expanded description of RNAseq data

The RNAseq profile of the wild-type strain, UTI89, revealed a total of 829 transcripts with significantly altered abundance across time, in response to the addition of ferric iron (**Supplementary File 1**, worksheet 1). Of these, 49 belonged to small non-coding RNAs, while 148 were hypothetical proteins (Supplementary File 1, worksheet 1; highlighted in light- and dark gray respectively). Of the remaining 632 transcripts, 81 belonged to tRNAs (n=50) and transcripts coding for tRNA modification- or translation-associated proteins (n=31) (**Supplementary File 1**, worksheet 1; highlighted in light pink). The non-coding RNA transcripts, as well as hypothetical and tRNA-/translation associated proteins were excluded from the heatmaps, along with 6 plasmid-associated transcripts unique to UTI89. The remaining 545 transcripts are depicted in heatmaps, according to the pathways they belong to (**Figure 3, 4**, **Supplementary files 1-2**).

Supplementary Figures and legends

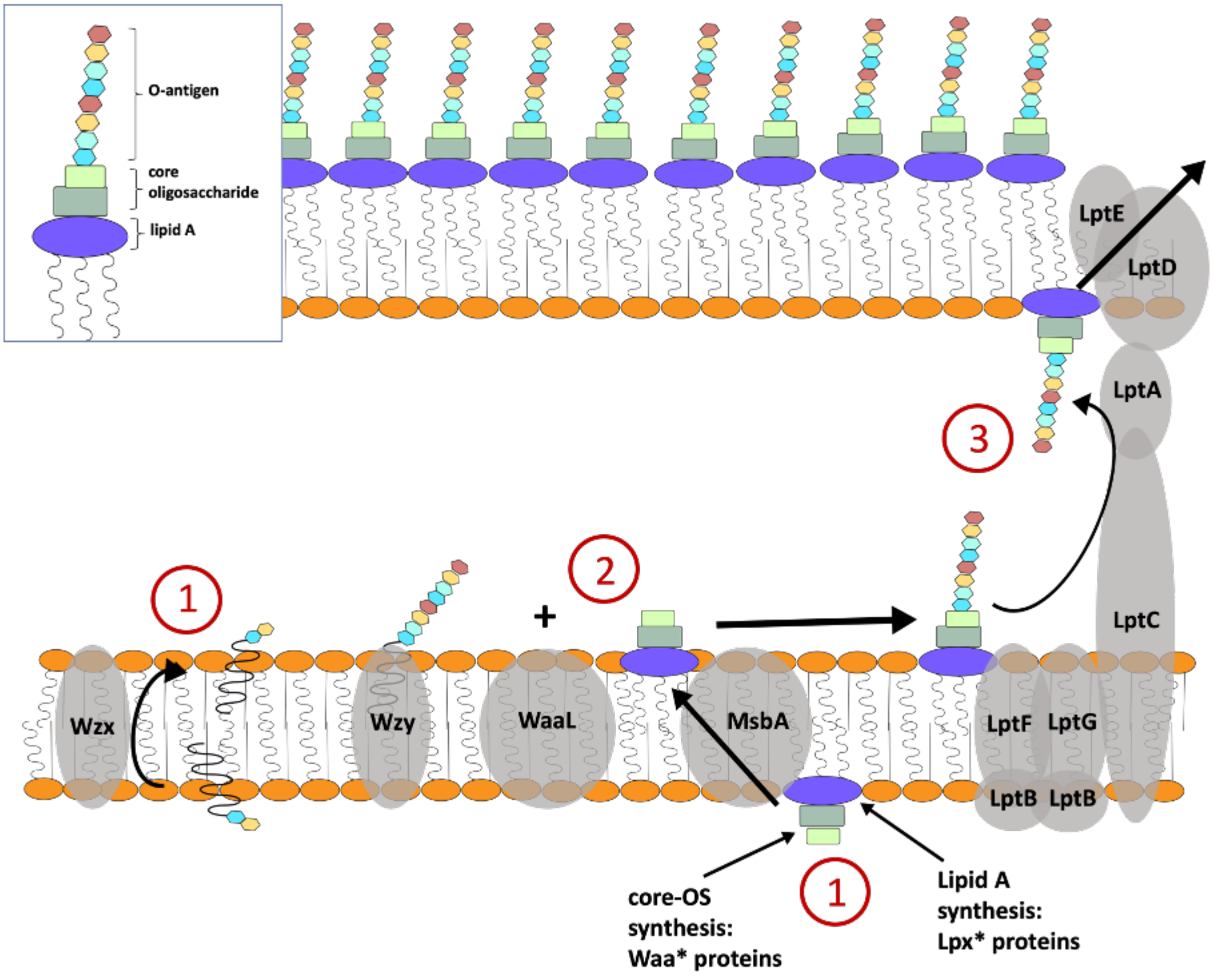

Figure S1: Cartoon depicts - in a simplified manner - the steps in lipopolysaccharide (LPS) biosynthesis in *Escherichia coli*. 1: lipid A is synthesized by the action of Lpx* proteins. Core oligosaccharide is synthesized by Waa* proteins. Together, the core-OS-lipid A molecule are flipped to the periplasm by MsbA. In parallel, O-antigen synthesis begins in the inner membrane on undecaprenyl phosphate Wzx flips the nascent O-antigen to the periplasm. Wzy polymerizes oligosaccharides onto the nascent chain maturing the O-antigen. 2: WaaL ligates the O-antigen to the core OS – lipid A molecule forming LPS. 3: LPS is translocated to the outer membrane by the Lpt system.

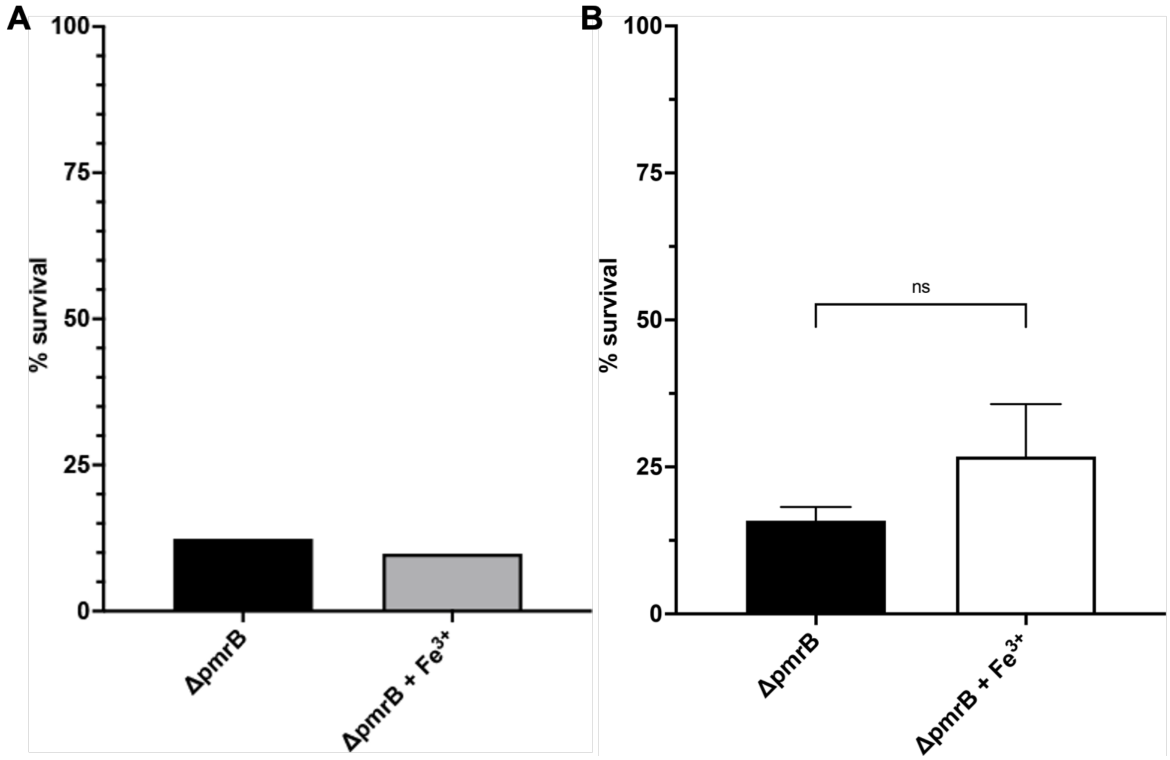

Figure S2: (A) Graph depicts the percent survival of UTI89Δ*pmrB* that was challenged with amikacin in a lone trial in the absence and presence of ferric iron for 60 minutes. (B) Graph depicts the percent survival of UTI89Δ*pmrB* (mean ± SEM, n = 3 biological repeats) with polymyxin B for 60 minutes in the presence and absence of ferric iron for 60 minutes. To determine statistical significance, an unpaired t-test was performed between each strain treated with ferric iron and its untreated isogenic control.

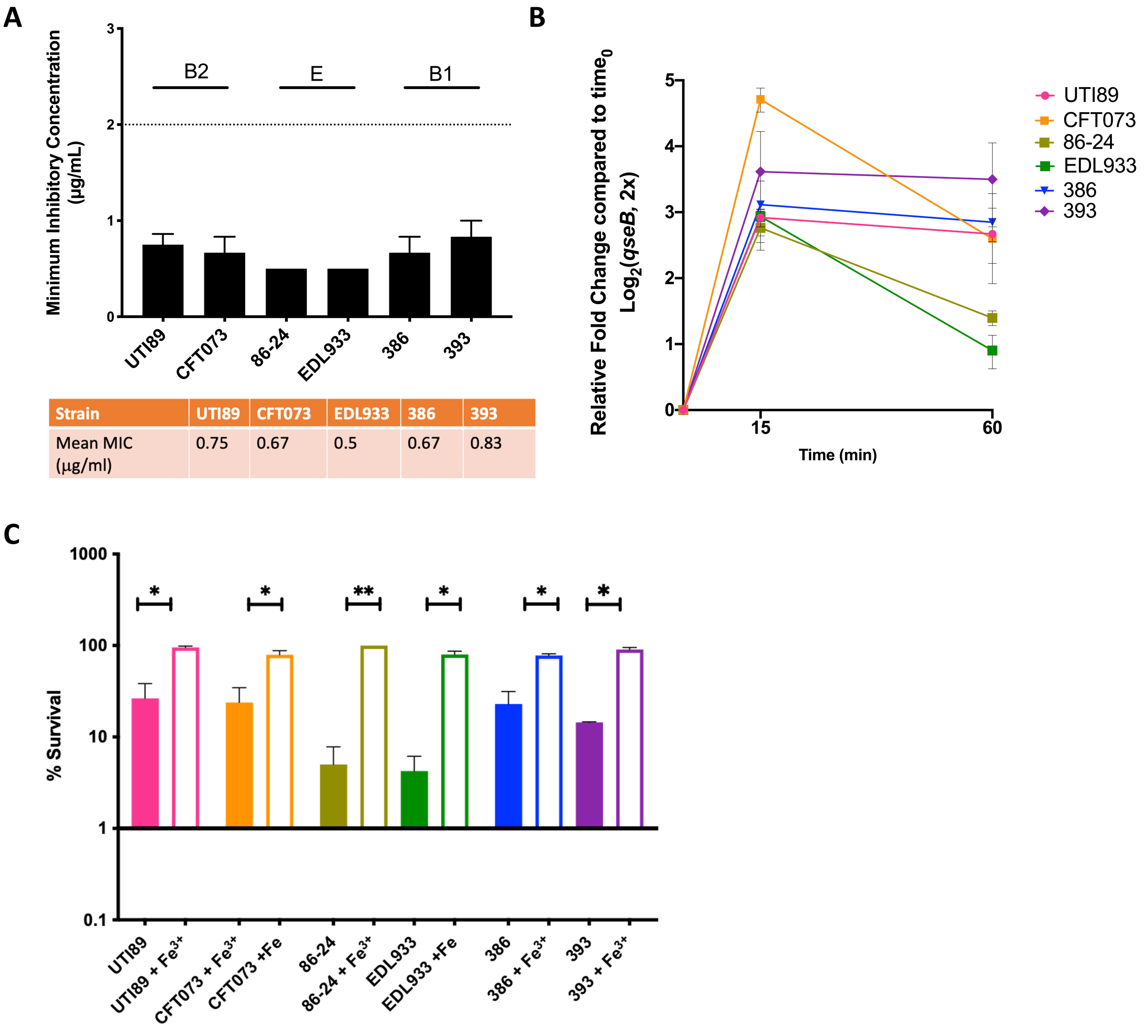

Strain	UTI89	CFT073	EDL933	386	393
Mean MIC (µg/ml)	0.75	0.67	0.5	0.67	0.83

Figure S3: (A) Graph and Table depict the polymyxin B minimum inhibitory concentration (MIC) determined for the strains of pathogenic *E. coli* used in our studies. These strains were selected so as to represent the most prevalent phylogenetic clades (mean ± SEM is shown, n = 3 biological repeats). (B) Graph depicts qPCR results tracking the activation of the *qse* operon following addition of ferric iron to the media. Briefly, cells were allowed to reach exponential growth phase. Cells were then collected before and at 15- and 60 minutes post addition of ferric iron. RNA was extracted, DNAse treated and reverse transcribed, as described in the methods section. The resulting cDNA was quantified, normalized for concentration and subjected to qPCR using TaqMan chemistry and a probe complementary *qseB* (See Table S1). Graph depicts log_2-fold change of *qseB* transcripts at each time point relative to the sample taken before stimulation (mean ± SEM, n = 3 biological repeats). (C) Graph depicts polymyxin survival of representative *E. coli* strains. (mean ± SEM, n = 3 biological repeats). To determine statistical significance, an unpaired t-test was performed between each strain treated with ferric iron and its untreated isogenic control.
*, $p < 0.05$; **, $p < 0.01$.

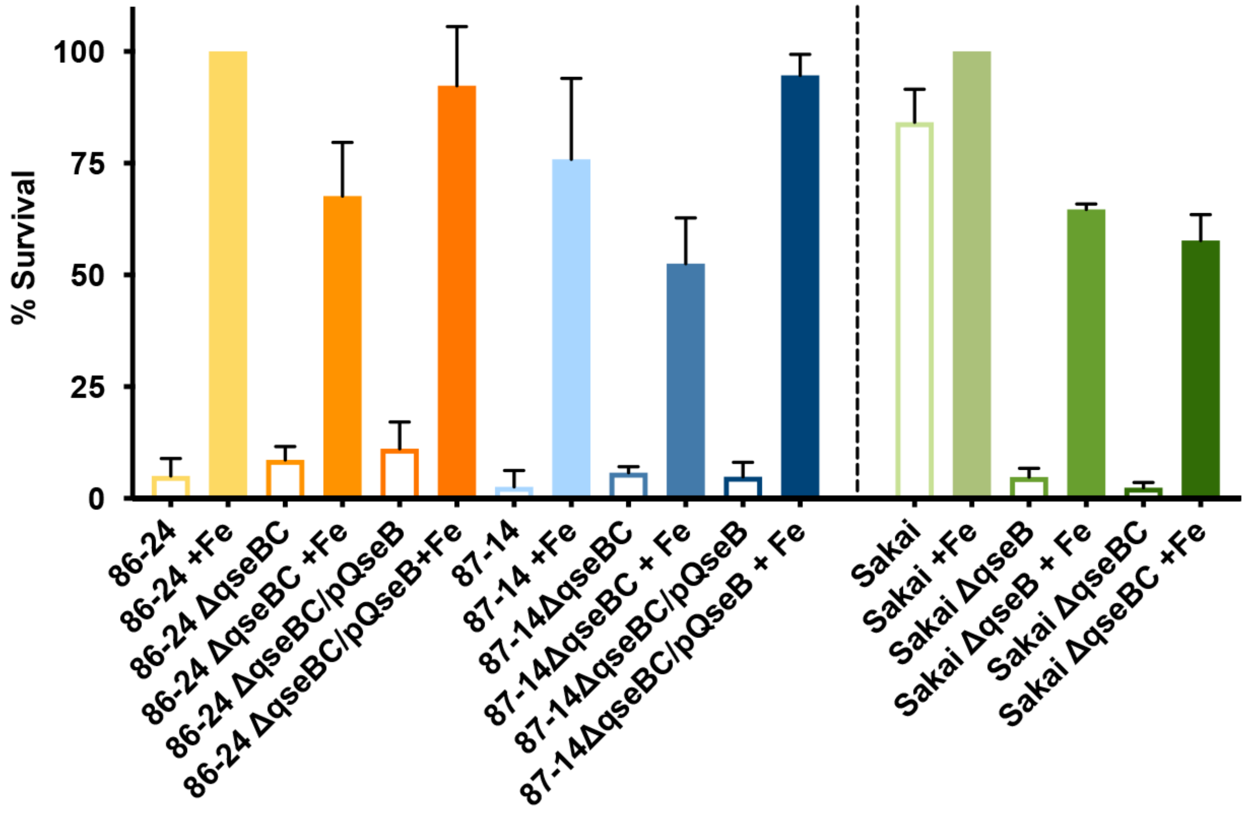

Figure S4: Graph depicts results of polymyxin B survival assays completed for representative EHEC strains and isogenic mutants. Cells were allowed to reach mid logarithmic growth phase in the presence or absence of ferric iron and normalized. Cells were then exposed to polymyxin at 2.5 µg/mL for one hour. At this time cells were serially diluted and plated to determine colony forming units per mL. To determine percent survival, cells exposed to polymyxin were compared to isogenic untreated controls from the same culture. (mean ± SEM, n = 3 biological repeats).

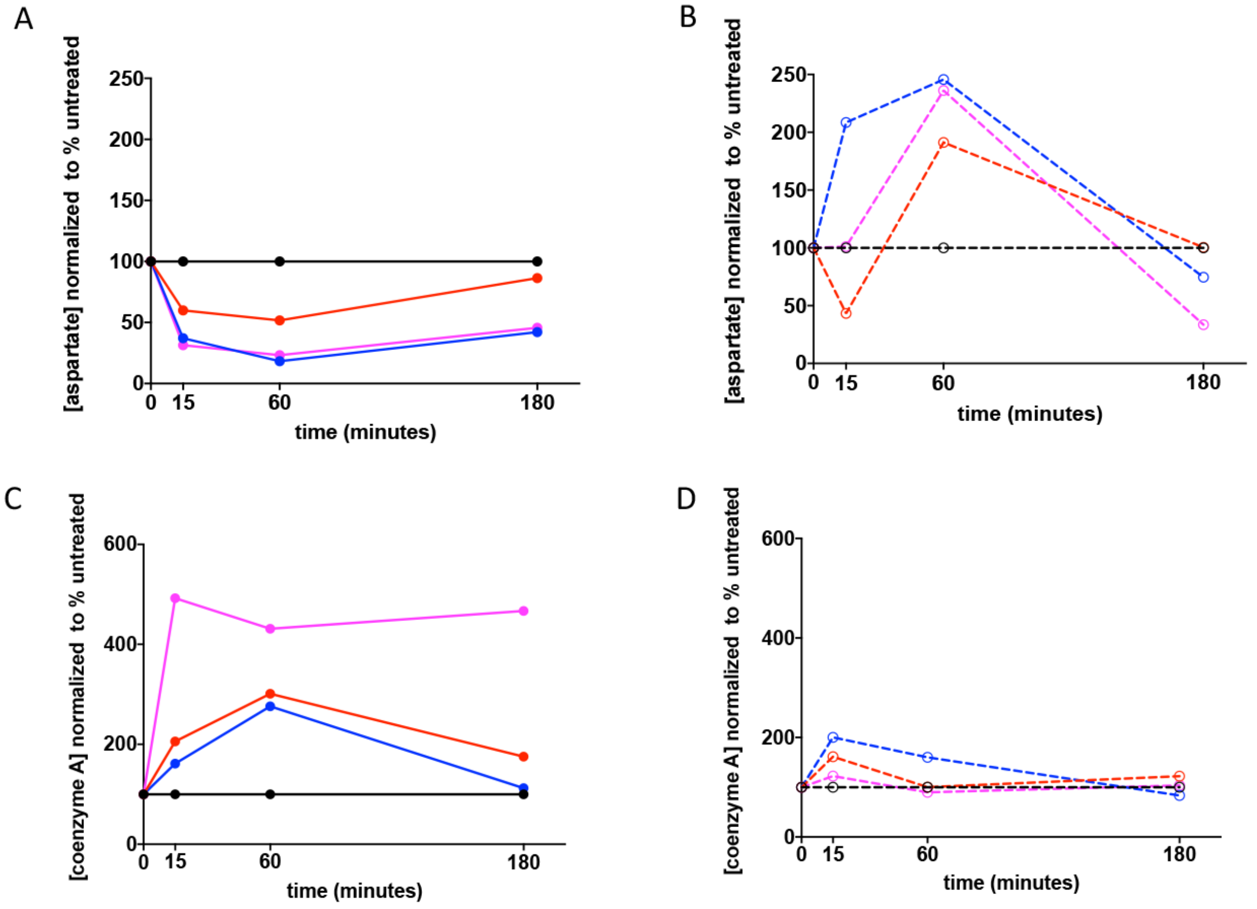

Figure S5: Graphs indicate metabolite abundance for aspartate and Co-enzyme A over time, in wild-type *E. coli* and isogenic mutants under different stimulation conditions. Measurements are normalized to a sample in which no additives or conditions were changed (black lines). Pink lines show measurements from samples in which ferric iron and polymyxin were added. Blue lines show measurements from samples in which only polymyxin was added. Red lines show measurements in which only ferric iron was added. **A-B)** Graphs depict glutamate measurements in wild-type UTI89 (A), or UTI89Δ*qseB* (B). **C-D)** Graphs depict aspartate measurements in wild-type UTI89 (C), or UTI89Δ*qseB* (D). Graphs are representative of at least three biological repeats.

Table S1: Strains, constructs, primers and probes used in this study

	SOURCE	IDENTIFIER
**Bacterial Strains**		
MG1655	Lab stock	N/A
CFT073	Lab stock	N/A
EDL933	ATCC	700927
86-24	Lab stock	N/A
ETEC 393	Rúgeles *et al* 2010	N/A
ETEC 386	Rúgeles *et al* 2010	N/A
UTI89	Lab stock	N/A
UTI89Δ*qseB*	Kostakioti *et al*	N/A
UTI89Δ*pmrB*	Guckes *et al* 2013	N/A
UTI89Δ*pmrA*	Guckes *et al* 2017	N/A
UTI89Δ*pmrA*Δ*pmrB*	Guckes *et al* 2017	N/A
UTI89Δ*ilvC*	This study	N/A
UTI89Δ*panD*	This study	N/A
**Plasmids**		
pTrc99A	Lab stock	N/A
pQseB-pTrcc99A	Kostakioti *et al*	N/A
pBAD-Myc-His-A	Lab stock	
pQseB-BAD-Myc-His-A	Lab stock	
**Oligonucleotides**		
GATGCGCGTGAAGGCCTGATTG	Guckes *et al* 2013	*gyrB*_qPCR_L
CACGGGCACGGGCAGCATC	Guckes *et al* 2013	*gyrB*_qPCR_R
CCTTATGATGCGGTGATCCTG G	Guckes *et al* 2013	*qseB*_qPCR_For
TCCCAGACGCAGCCCTTCTA	Guckes *et al* 2013	*qseB*_qPCR_Rev
6FAMTGCGCGAATGGCGA	Guckes *et al* 2013	*qseB*_qPCR_probe
VICACGAACTGCTGGCGGA	Guckes *et al* 2013	*gyrB*_qPCR_probe
CGAAGATGCTGCCCATG	This study	*yfbE*_qPCR_For
GCTGGCGGGCAAGATT	This study	*yfbE*_qPCR_Rev
6FAMAGGGCGACATATTGGCGCAAAAGG TACTGCT	This study	*yfbE*_qPCR_probe
TACATTGAAGGTGATGGAATCGGTGTAG ATGTAACCCCAGCCATGCTGAAGTGTAG GCTGGAGCTGCTTC	This study	*icdA*_knockout_For
GGCATTGATTGCGCCTTCCATACCTTTAA CAATCAGGTCAGCCGCTTCAGCATATGA ATATCCTCCTTAG	This study	*icdA*_knockout_Rev
CGCTCGAAGGAGAGGTGAAT	This study	*icdA*_knockout_test_For
GCCCGTTTCAATATTTAACACATG	This study	*icdA* _knockout_test_Rev
ATCGCCCGTCTCAAAGGTATTAATGAAGA TCTCTCGTTAGAAGAAGTTGCGTGTAGG CTGGAGCTGCTTC	This study	*panD*_knockout_For
TAGTCTGACCTCTTCTACTGCATGATTAG CACTTTTCGTCAGGATTAAACCATATGAA TATCCTCCTTAG	This study	*panD*_knockout_Rev
GCTATGACCGCCAGAAACATG	This study	*panD*_knockout_test_For
GCGGTGAAATATCCCTGCGT	This study	*panD*_knockout_test_Rev
